# 意义未明的单克隆丙种球蛋白血症的临床特征及疾病进展相关因素分析

**DOI:** 10.3760/cma.j.issn.0253-2727.2023.02.009

**Published:** 2023-02

**Authors:** 艾 关, 恺妮 沈, 路 张, 欣欣 曹, 薇 苏, 道斌 周, 剑 李

**Affiliations:** 1 中国医学科学院、北京协和医学院北京协和医院血液内科，北京 100730 Department of Hematology, Peking Union Medical College Hospital, Chinese Academy of Medical Sciences & Peking Union Medical College, Beijing 100730, China; 2 中国医学科学院、北京协和医学院北京协和医院检验科，北京 100730 Department of Clinical Laboratory, Peking Union Medical College Hospital, Chinese Academy of Medical Sciences & Peking Union Medical College, Beijing 100730, China

**Keywords:** 意义未明单克隆丙种球蛋白病, 疾病恶化, Monoclonal gammopathy of undetermined significance, Disease progression

## Abstract

**目的:**

探究我国意义未明的单克隆丙种球蛋白血症（MGUS）患者的临床特征及疾病进展相关因素。

**方法:**

回顾性分析2004年1月至2022年1月北京协和医院确诊的1 037例MGUS患者的临床资料。

**结果:**

1 037例患者中，男636例（61.3％），中位诊断年龄58（18～94）岁，中位血清单克隆免疫球蛋白（M蛋白）水平为2.7（0～29.4）g/L。IgG型、IgA型、IgM型、IgD型、轻链型分别为380例（59.7％）、143例（22.5％）、103例（16.2％）、4例（0.6％）、6例（0.9％）。血清游离轻链比例（sFLCr）异常患者171例（31.9％）。按照梅奥危险分层，低危、中低危、中高危、高危组患者分别为254例（59.5％）、126例（29.5％）、43例（10.1％）、4例（0.9％）。中位随访47（1～204）个月后，34例（4.3％）发生疾病进展，22例（2.8％）死亡，进展率为1.06（0.99～1.13）/100人年。IgM-MGUS的进展率显著高于非IgM-MGUS（2.87/100人年对0.99/100人年，*P*＝0.002）。在非IgM-MGUS组患者中，梅奥分层为低危、中低危、中高危组患者的进展率依次为0.32（0.25～0.39）/100人年、1.82（1.55～2.09）/100人年和2.71（1.93～3.49）/100人年，差异有统计学意义（*P*＝0.005）。

**结论:**

与非IgM-MGUS相比，IgM-MGUS的疾病进展风险更高。梅奥危险分层模型适用于中国非IgM-MGUS患者。

## Abstract (en)

**Objective:**

To analyze the clinical presentation and progression risk factors of patients with monoclonal gammopathy of undetermined significance（MGUS）in China.

**Methods:**

We retrospectively assessed the clinical features and disease progression of 1 037 patients with monoclonal gammopathy of undetermined significance between January 2004 and January 2022 at Peking Union Medical College Hospital.

**Results:**

A total of 1 037 patients were recruited in the study, including 636 males（63.6％）, with a median age of 58（18–94）years. The median concentration of serum monoclonal protein was 2.7（0–29.4）g/L. The monoclonal immunoglobulin type was IgG in 380 patients（59.7％）, IgA in 143 patients（22.5％）, IgM in 103 patients（16.2％）, IgD in 4 patients（0.6％）, and light chain in 6 patients（0.9％）. 171 patients（31.9％）had an abnormal serum-free light chain ratio（sFLCr）. According to the Mayo Clinic model for risk of progression, the proportion of patients in the low-risk, medium-low-risk, medium-high risk, and high-risk groups were 254（59.5％）, 126（29.5％）, 43（10.1％）, and 4（0.9％）, respectively. With a median follow-up of 47（1–204）months, 34 of 795 patients（4.3％）had disease progression, and 22（2.8％）died. The overall progression rate was 1.06（0.99–1.13）/100 person-years. Patients with non-IgM MGUS have a markedly higher disease progression rate per 100 person-years than IgM-MGUS（2.87/100 person-years *vs* 0.99/100 person-years, *P*＝0.002）. The disease progression rate per 100 person-years in non-IgM-MGUS patients of Mayo classification low-risk, medium-low risk and medium-high risk groups were 0.32（0.25–0.39）/100 person-years, 1.82（1.55–2.09）/100 person-years, and 2.71（1.93–3.49）/100 person-years, which had statistically difference（*P*＝0.005）.

**Conclusion:**

In comparison to non-IgM-MGUS, IgM-MGUS has a greater risk of disease progression. The Mayo Clinic progression risk model applies to non-IgM-MGUS patients in China.

意义未明的单克隆丙种球蛋白血症（MGUS）是一类常见的癌前病变[1]。临床常用的MGUS危险度分层模型是梅奥诊所提出的基于血清M蛋白水平、M蛋白类型和血清游离轻链比例（sFLCr）三个因素的疾病进展风险分层模型[2]。目前国内尚缺少大宗MGUS患者的临床特征及疾病进展研究，因此，本研究回顾性分析了单中心1 073例MGUS患者的临床资料，以探究中国MGUS患者的临床特征及疾病进展的危险因素。

## 病例与方法

1. 病例：共纳入1 037例2004年1月至2022年1月在北京协和医院确诊的MGUS患者。所有患者均符合以下诊断标准：①血清M蛋白<30 g/L；②骨髓单克隆浆细胞<10％；③不存在浆细胞增殖相关的终末器官损害及SLiM表现[1]。收集患者的临床资料，包括人口学资料、实验室检查、生存及疾病进展情况等。IgM型MGUS定义为符合以下标准：①血清存在IgM型M蛋白；②骨髓内无淋巴浆细胞浸润；③缺乏相关症状或器官损害[1]。

2. 梅奥危险分层[2]：梅奥危险分层模型包括3个危险因素：血清M蛋白>15 g/L、M蛋白分类为非IgG型、sFLCr异常（定义为<0.26或>1.65）。按照危险因素数目对患者进行分层：低危：0个危险因素；中低危：1个危险因素；中高危：2个危险因素；高危：3个危险因素。

3. 随访：通过门诊病历系统或电话进行随访，末次随访时间为2022年3月31日。疾病进展定义为冒烟型骨髓瘤、多发性骨髓瘤（MM）、华氏巨球蛋白血症（WM）、轻链型淀粉样变、B细胞非霍奇金淋巴瘤（B-NHL）、孤立性浆细胞瘤等淋巴细胞或浆细胞肿瘤，其中IgM型MGUS进展为WM需满足骨髓内出现淋巴浆细胞浸润，且存在WM相关临床症状。无进展生存（PFS）期定义为自患者确诊至疾病进展或因任何原因死亡的时间。总生存（OS）期定义为自患者确诊至因任何原因死亡或末次随访的时间。

4. 统计学处理：采用SPSS 22.0软件、R 4.0.2、Stata 16进行统计学分析，采用R 4.0.2作图。分类变量用例数（百分比）描述，连续变量采用中位数（范围）描述。人年进展率的计算公式为随访期内进展病例数/∑［患者随访时长（年）］。利用泊松分布计算95％置信区间，利用iri函数（Stata 16）进行进展率差异检验[3]。所有检验为双侧检验，*P*<0.05为差异有统计学意义。

## 结果

1. 一般特征：共纳入1 037例患者，其中男636例（61.3％），中位诊断年龄58（18～94）岁，471例（45.4％）为体检发现。中位血清M蛋白水平为2.7（0～29.4）g/L，其中<5 g/L、≥5 g/L且≤10 g/L、>10 g/L且≤15 g/L、>15 g/L者分别为749例（74.4％）、150例（14.9％）、59例（5.9％）、48例（4.8％）。在有血清M蛋白类型数据的636例患者中，IgG型380例（59.7％）、IgA型143例（22.5％）、IgM型103例（16.2％）、IgD型4例（0.6％）、单纯轻链型6例（0.9％）。血清轻链类型κ型327例（50.4％）、λ型322例（49.6％）。背景免疫球蛋白降低（IgG降低定义为IgG<7 g/L，IgA降低定义为IgA<0.7 g/L，IgM降低定义为IgM<0.4 g/L）数量为0个、1个、2个、3个的患者依次为430例（76.1％）、109例（19.3％）、25例（4.4％）、1例（0.2％）。高IgM血症（IgM>2.3 g/L）患者77例（14.2％）。sFLCr异常患者171例（31.9％）。427例患者具有完整的梅奥危险分层数据，其中低危组254例（59.5％）、中低危组126例（29.5％）、中高危组43例（10.1％）、高危组4例（0.9％）。

2. 进展和生存分析：795例患者具有长期随访信息，中位随访时间为47（1～204）个月，总计随访3 267.0人年。共有34例（4.3％）患者出现疾病进展。其中，4例（11.8％）进展为冒烟型骨髓瘤，11例（32.4％）进展为MM，5例（14.7％）进展为WM，7例（20.6％）进展为轻链型淀粉样变，6例（17.6％）进展为B-NHL，1例（2.9％）进展为孤立性浆细胞瘤。中位PFS期尚未达到。死亡22例（2.8％），其中2例（9.1％）死于疾病进展，20例（90.9％）死于其他疾病，中位OS期尚未达到，总体死亡率为0.70（0.64～0.76）/100人年。

在非IgM-MGUS组（439例）中，3例（17.6％）进展为冒烟型骨髓瘤，8例（47.1％）进展为MM，4例（22.4％）进展为轻链型淀粉样变，1例（5.6％）进展为B-NHL，1例（5.6％）进展为孤立性浆细胞瘤。IgM-MGUS组（83例）中，5例（45.4％）进展为WM，5例（45.4％）进展为B-NHL，1例（9.1％）进展为轻链型淀粉样变。非IgM-MGUS组和IgM-MGUS组患者的死亡率分别为0.41（0.35～0.47）/100人年和2.49（2.15～2.82）/100人年（*P*<0.001）。

3. 进展相关危险因素分析：MGUS患者的总体进展率为1.06（0.99～1.13）/100人年。梅奥分层低危、中低危、中高危、高危组患者的进展率分别为0.31/100人年、2.25/100人年、2.95/100人年、22.57/100人年（*P*<0.001）。IgM-MGUS组的进展率显著高于非IgM-MGUS组［2.87（2.51～3.24）/100人年对0.99（0.89～1.08）/100人年，*P*＝0.002］。

在非IgM-MGUS组患者中，梅奥分层为低危、中低危、中高危组患者的进展率依次为0.32（0.25～0.39）/100人年、1.82（1.55～2.09）/100人年和2.71（1.93～3.49）/100人年（*P*＝0.005）（表1）。在IgM-MGUS组患者中，血清M蛋白>15 g/L与≤15 g/L患者的疾病进展率差异无统计学意义（*P*＝0.131），sFLCr异常与正常患者疾病进展率的差异无统计学意义（*P*＝0.386），不同梅奥危险分层患者的疾病进展率差异无统计学意义（*P*＝0.445）（表1）。

**表1:** 非IgM-MGUS组与IgM-MGUS组疾病进展的危险因素分析

因素	非IgM-MGUS（439例）			IgM-MGUS（83例）		
	进展率/100人年（95%*CI*）	*RR*（95%*CI*）	*P*值	进展率/100人年（95%*CI*）	*RR*（95%*CI*）	*P*值
年龄			0.052			0.357
≤65岁	1.24（1.12～1.36）	1		3.09（2.55～3.64）	1	
>65岁	0.39（0.28～0.50）	0.32（0.26～0.39）		2.45（1.98～2.93）	0.79（0.51～1.22）	
性别			0.225			0.193
女	1.20（1.04～1.36）	1		3.80（3.11～4.50）	1	
男	0.83（0.71～0.94）	0.69（0.57～0.83）		2.22（1.82～2.62）	0.58（0.37～0.91）	
血清M蛋白			<0.001			0.131
≤15 g/L	0.76（0.67～0.84）	1		3.07（2.60～3.54）	1	
>15 g/L	3.28（2.58～3.98）	4.32（2.90～6.42）		12.64（8.62～16.67）	4.12（1.29～13.18）	
sFLCr			0.025			0.386
正常	0.62（0.52～0.71）	1		3.51（2.74～4.28）	1	
异常	1.81（1.56～2.07）	2.94（2.35～3.68）		4.44（3.67～5.20）	1.26（0.73～2.18）	
M蛋白类型			0.003		
IgG型	0.72（0.63～0.82）	1		−	−	
IgA型	2.97（2.65～3.30）	4.11（3.31～5.10）		−	−	
梅奥危险分层			0.005			0.445
低危	0.32（0.25～0.39）	1		−	−	
中低危	1.82（1.55～2.09）	5.71（4.52～7.22）		3.51（2.74～4.28）	1	
中高危	2.71（1.93～3.49）	8.50（5.20～13.90）		3.18（2.48～3.88）	0.91（0.51～1.60）	
高危	−	−		25.42（18.44～32.41）	7.24（1.71～30.71）	

**注** MGUS：意义未明的单克隆丙种球蛋白血症；sFLCr：血清游离轻链比例；−：无数据

## 讨论

MGUS是一种较为常见的癌前疾病，在欧美地区50岁以上人群中的发病率约为3.2％[4]。本中心基于健康人群体检数据的MGUS流行病学调查发现，50岁以上人群中MGUS的患病率约为1.11％，低于欧美人群[5]。MGUS可进展为恶性浆细胞疾病或淋巴细胞增殖性疾病，既往研究表明MGUS患者的进展率为1.0（0.9～1.2）/100人年，中位生存期约为20年[2]。本研究中患者的进展率为1.06（0.99～1.13）/100人年，与既往研究基本一致。

基线临床特征可以预测MGUS的进展风险。2007年Pérez-Persona等[6]提出了PETHEMA模型，此模型需要骨髓流式细胞术检查结果，而MGUS患者往往不进行骨髓检查，因此该模型的临床应用价值较低。2014年瑞士一项纳入728例MGUS患者的研究表明，正常免疫球蛋白减少可能与疾病进展相关[7]–[8]。2018年Kyle等[2]总结了梅奥诊所随访20年的1 384例患者的临床数据，提出血清M蛋白>15 g/L、M蛋白类型为IgM型和sFLCr异常是MGUS进展的危险因素，根据危险因素数目进行分层后，低危组、中低危组、中高危组、高危组的疾病进展率分别为0.4/100人年、1.0/100人年、1.7/100人年和3.6/100人年（*P*<0.001）。本研究结果与梅奥诊所报告的危险分层趋势基本一致，其中高危组疾病进展率与既往研究相比差异较大，可能是由于该组仅有4例患者导致统计偏倚较高。

MGUS的疾病进展类型与M蛋白类型相关。不同M蛋白类型MGUS的临床特征和受累细胞具有显著差异，因此其进展模式并不相同[9]。非IgM型MGUS通常起病于经历了类别转换的成熟浆细胞，具有进展为冒烟型骨髓瘤并进一步发展为MM的倾向。而IgM型MGUS的受累细胞类型通常为未经过类别转换的淋巴细胞，因此倾向于进展为淋巴瘤或WM，进展风险相对更高，还有少数也可进展为轻链型淀粉样变[10]。轻链型MGUS更容易进展为轻链型MM、轻链型淀粉样变或轻链沉积病[11]–[12]。本研究同样表明，IgM-MGUS的进展率高于非IgM-MGUS，MM（100％）及冒烟型骨髓瘤（100％）均进展自非IgM-MGUS，WM（100％）和大部分B-NHL（83.3％）均进展自IgM-MGUS患者。

在进展危险因素方面，本研究同样显示，不同梅奥危险分层的非IgM-MGUS患者的进展率具有显著差异。但M蛋白水平、sFLCr和梅奥危险分层均与IgM-MGUS组的疾病进展无关。2017年Andrade-Campos等[13]的一项研究发现受累和非受累血清重/轻链比值异常增高与IgM-MGUS进展相关。IgM-MGUS的其他进展危险因素还包括CXCR4、MYD88等基因突变[14]，本研究证实了IgM-MGUS和非IgM-MGUS在临床特征和进展模式上有一定差别，IgM-MGUS的疾病进展相关因素仍有待进一步探究。

综上，IgM-MGUS患者的长期疾病进展风险较非IgM-MGUS患者更高，容易进展为WM和B-NHL。梅奥诊所提出的梅奥危险分层模型适用于非IgM-MGUS患者，但不适用于IgM-MGUS患者。
